# Previous induced abortion among young women seeking abortion-related care in Kenya: a cross-sectional analysis

**DOI:** 10.1186/s12884-016-0894-z

**Published:** 2016-05-14

**Authors:** Caroline W. Kabiru, Boniface A. Ushie, Michael M. Mutua, Chimaraoke O. Izugbara

**Affiliations:** African Population and Health Research Center, 2nd Floor APHRC Campus, Manga Close Off Kirawa Road, P.O. Box 10787-00100, Nairobi, Kenya; John C Caldwell Population, Health and Development Visiting Fellow, the National Centre for Epidemiology & Population Health (NCEPH) and School of Demography, Australian National University, 9 Fellows Road, Acton, ACT 2601 Australia; Institute of Child Health, College of Medicine, University of Ibadan, Ibadan, Nigeria; School of Public Health, University of Witwatersrand, Wits Education Campus, 27St. Andrews Road, Parktown, 2193 Johannesburg, South Africa

**Keywords:** Multiple Induced Abortions, Young Women, Sexual and Reproductive Health, Contraception, Unintended Pregnancy, Sub-Saharan Africa, Kenya

## Abstract

**Background:**

Unsafe abortion is a leading cause of death among young women aged 10–24 years in sub-Saharan Africa. Although having multiple induced abortions may exacerbate the risk for poor health outcomes, there has been minimal research on young women in this region who have multiple induced abortions. The objective of this study was therefore to assess the prevalence and correlates of reporting a previous induced abortion among young females aged 12–24 years seeking abortion-related care in Kenya.

**Methods:**

We used data on 1,378 young women aged 12–24 years who presented for abortion-related care in 246 health facilities in a nationwide survey conducted in 2012. Socio-demographic characteristics, reproductive and clinical histories, and physical examination assessment data were collected from women during a one-month data collection period using an abortion case capture form.

**Results:**

Nine percent (*n* = 98) of young women reported a previous induced abortion prior to the index pregnancy for which they were receiving care. Statistically significant differences by previous history of induced abortion were observed for area of residence, religion and occupation at bivariate level. Urban dwellers and unemployed/other young women were more likely to report a previous induced abortion. A greater proportion of young women reporting a previous induced abortion stated that they were using a contraceptive method at the time of the index pregnancy (47 %) compared with those reporting no previous induced abortion (23 %). Not surprisingly, a greater proportion of young women reporting a previous induced abortion (82 %) reported their index pregnancy as unintended (not wanted at all or mistimed) compared with women reporting no previous induced abortion (64 %).

**Conclusions:**

Our study results show that about one in every ten young women seeking abortion-related care in Kenya reports a previous induced abortion. Comprehensive post-abortion care services targeting young women are needed. In particular, post-abortion care service providers must ensure that young clients receive contraceptive counseling and effective pregnancy prevention methods before discharge from the health care facility to prevent unintended pregnancies that may result in subsequent induced abortions.

## Background

An estimated 6.4 million abortions occurred in Africa in 2008 [1]. Almost all of these abortions (97 %) [1] were unsafe—that is, they were performed under conditions that fail to meet minimal medical standards and/or by untrained persons [2]. Unsafe abortion is a leading cause of morbidity and mortality in many sub-Saharan African countries [3]. In Kenya, an estimated 35 % of maternal deaths are attributed to unsafe abortions [4, 5]. Young women aged 10–24 years are at heightened risk for unsafe abortion due to their high vulnerability to unintended pregnancies [6–9].

Kenya is classified as a Category II country under the World’s Abortion Laws [10]. The country’s laws only permit abortion to protect a woman’s life and health. Kenya’s restrictive abortion laws and the high stigma around premarital sexual activity, which limits access to sexual and reproductive health services and effective contraception, mean that many young women with unintended pregnancies resort to unsafe abortion. Not surprisingly, young females form the bulk of women who present for care following unsafe abortion in Kenya [11]. Young women are also more likely to present with severe complications and to receive lower quality treatment [11]. Further, limited uptake of effective contraceptive methods after an abortion [11] may place many young women at risk for subsequent unintended pregnancies and abortion.

Data on multiple induced abortions among adolescents and young women are limited globally. In one of the few studies on multiple induced abortions among young women, Collier [12] found an increase in the proportion of adolescent women (under 20 years of age) in the United Kingdom reporting multiple induced abortions over the 17-year period between 1991 and 2007 (from 8 to 13 %). Tietze [13] posits that the proportion of women reporting multiple induced abortions would increase with age and parity because of the increased number of women who have had a first abortion and are at risk of having a subsequent abortion. However, studies assessing the association between age and the likelihood of having multiple induced abortions have generated mixed results. Some studies have found that younger women have a lower likelihood of reporting multiple induced abortions compared with older women [14, 15]. Conversely, a prospective study in Finland found that women younger than 20 years were significantly more likely to request a subsequent abortion compared with those over the age of 25 years [16].

Beyond age differentials, previous studies on multiple induced abortions have found differences between women based on socio-demographic characteristics (e.g., socio-economic status [17, 18], level of education [15, 17]), relationship status [18], behavioral factors (e.g., age at first sexual intercourse [17], alcohol and cigarette use [16, 19], number of sexual partners [17], contraceptive use [16, 17, 20]), as well as obstetric factors (e.g., parity [15, 16, 18, 21], pregnancy intentions [15], a history of previous abortions [16]). The bulk of these studies were conducted in countries with relatively liberal abortion laws. While these studies provide useful insights about multiple induced abortions among young women, little is known about the prevalence of and factors associated with multiple induced abortions among young women in settings where the majority of abortions may occur outside formal health care settings because of restricted safe abortion service provision [10]. In this study, we examine the factors associated with the likelihood of reporting a previous induced abortion among young females aged 10–24 years receiving abortion-related care in Kenya.

## Methods

### Data and procedures

This study draws on secondary data from a study on the magnitude and incidence of induced abortion that was conducted in 328 nationally-representative health facilities in Kenya [11]. Health facilities were selected using a stratified random sampling procedure based on the Kenya Essential Package for Health classification of six levels of preventive and curative health services. A health care facility level is a description of functionality as defined by the Kenyan Ministry of Health. Level 1 is the lowest level of care, while Level 6 is the premier level of health care in Kenya [22]. In Kenya, only Level 2–6 facilities (*N* = 2,838) can provide post-abortion care services.

A sample of 350 facilities was considered adequate to detect a 10 % difference in the severity of abortion-related complications at the regional level at 80 % power. The minimum sample size of facilities was computed using Epi-Info. Data weights were computed based on the product of the probability of a facility being selected in the sample and the probability of the facility participating in the survey. From a national frame of *N* = 2,828 facilities, each of the 350 facilities had a probability p_1_ = 0.1233 of being selected and participating. The sample of facilities was selected from a master list of all relevant health care facilities and stratified based on the level and geo-political region. All Level 5 and 6 facilities and a random sample of Level 2–4 facilities were included. Among the 350 sampled facilities, 328 facilities (94 %) participated, giving each facility a probability p_2_ = 0.937 of participating. Therefore, each facility had a probability p_12_ = 0.116 of being selected and participating in the survey. This probability was allowed to vary according to facility level and geographic location. The data weights used in the analysis were the reciprocals of these probabilities (i.e. w_i_ = 1/p_12_).

At the client-level, the main study aimed to achieve a sample of 2,500 female clients of all ages for estimation of abortion complication rates, assuming an average of 7.1 women per facility. For our analyses, we needed a sub-sample of at least 80 women in each stratum, to detect a 10 % difference in our sample. Thus, with 9 % of our sample reporting a previous induced abortion (the outcome variable), a sample of 1,378 women maintained all our analyses above 99 % power. The power computation for the secondary analysis was conducted using Stata’s *sampsi* command.

Data were collected for all females presenting for abortion-related treatment during a one-month data collection period (April-May 2012). Data were collected using an abortion case capture form, which gathered information on socio-demographic characteristics, reproductive and clinical histories, and physical examination assessments. Facility-based health providers were trained to collect the data. A total of 1,383 young women aged 12–24 years presented for abortion-related care in 246 facilities out of the 328 participating facilities. Of these, 1,378 (99.6 %) had complete history data on previous induced abortions and comprised the final analytical sample for the study.

### Variables

The primary outcome variable, previous history of induced abortion, was based on responses to a question asking the young woman the number of previous induced abortions excluding the one she was currently seeking care for. Ninety-eight young women reported a previous induced abortion. We also measured the following socio-demographic characteristics that in previous studies [16, 21, 23] have been found to be associated with the likelihood of reporting multiple abortions: age, residence (rural versus urban), highest level of education attained, religious affiliation, and occupation. Our multivariable analysis included all the socio-demographic characteristics as well as three reproductive variables (parity, type of contraception used at the time of the index pregnancy, and pregnancy desire) that have been found to be associated with the likelihood of reporting multiple abortions in previous studies [16, 18, 21]

### Ethical considerations

The study protocol was reviewed and approved by the Ethical Review Boards of the Kenya Medical Research Institute, the University of Nairobi/ Kenyatta National Hospital, Moi University Teaching and Referral Hospital, Kenya, and Aga Khan University, Kenya. The Ministries of Public Health and Sanitation and Medical Services in Kenya and the Institutional Review Board of the Guttmacher Institute also reviewed and approved the study protocol. Verbal informed consent was obtained from all women presenting for care (or their caregivers) prior to data collection.

### Analyses

Descriptive statistics of the respondents’ socio-demographic characteristics, reproductive and clinical histories, as well as diagnosis and clinical examination findings (e.g., severity of complications) were computed. Data were weighted to account for the survey design. We expected that adolescents and young women seeking care in one facility would have similar characteristics. Bivariate and multivariable logistic regression models were therefore performed accounting for the sampling design (individuals nested within facilities stratified by level) using the *svy* set of commands available in Stata Version 13. For each socio-demographic and reproductive variable, we ran logistic regression models to generate unadjusted odds ratios for bivariate associations with the outcome variable. We then ran a model including all the socio-demographic variables to generate odds ratios adjusted for other socio-demographic variables. Finally, we ran a model with all the socio-demographic and reproductive variables to generate odds ratios adjusted for socio-demographic and reproductive variables. *P*-values of less than 0.05 based on adjusted Wald tests for differences between odds ratios were considered statistically significant.

## Results

Table 1 summarizes respondents’ socio-demographic characteristics by previous history of induced abortion. Adolescents (12–19 years) comprised about a third of the young women who presented for abortion-related care. Three out of every five young women receiving abortion-related care were rural residents. Twenty-seven percent of young women had no education or had not completed primary school. Eleven percent had a post-secondary level of education. Majority of the respondents (87 %) reported Christian religious affiliation. Twenty-seven percent of the young women were students at the time they presented for care while 33 % were employed either as skilled or unskilled workers.

**Table 1:** Socio-demographic profile (%) of young women (12–24 years) seeking abortion care

	No previous induced abortion	Previous induced abortion	All young women
	*n* = 1,280 (91 %)	*n* = 98 (9 %)	*N* = 1,378 (100 %)
Age group			
10–19 years	34	36	34
20–24 years	66	64	66
Residence*			
Urban	38	64	40
Rural	62	36	60
Education^†^			
No education/incomplete primary	29	11	27
Complete primary	17	23	17
Incomplete secondary	20	27	21
Complete secondary	23	30	23
Post-secondary	11	9	11
Marital status			
Never married	50	63	51
Currently/formerly married	50	37	49
Religion*			
Roman Catholic	19	27	19
Other Christian	68	64	68
Muslim	11	2	10
Other/No religion	2	8	2
Occupation*			
Farmer/unskilled laborer	20	32	21
Skilled/clerical/sales/services	12	19	12
Student	26	36	27
Housewife	27	4	25
Unemployed/other	16	9	16

^†^ *p* < 0.10; *p < 0.05 for differences between young women based on previous history of induced abortion based on chi-square tests

Nine percent (*n* = 98) of young women reported at least one previous induced abortion before the index pregnancy. Statistically significant differences by previous history of induced abortion were observed for area of residence, religion and occupation. Specifically, although young women living in urban areas only comprised 40 % of the total number of young women seeking abortion-related care, urban dwellers comprised almost two-thirds of women reporting a previous induced abortion. Students comprised 27 % of the total sample of young women but represented 36 % of those reporting a previous induced abortion.

Table 2 summarizes the reproductive characteristics of young women seeking abortion care by previous history of induced abortion. A greater proportion of young women reporting a previous induced abortion (47 %) reported the use of a method of contraception at the time of the index pregnancy compared with those reporting no previous induced abortion (23 %). However, a large proportion of contraceptive users reporting a previous induced abortion were using methods known to have high failure rates [24]. Specifically, 42 % of contraceptive users who stated that they had a previous abortion reported the use of emergency contraception while 12 % reported the use of the rhythm method, withdrawal or lactational amenorrhea (not shown). A greater proportion of young women who stated that they had a previous induced abortion (82 %) reported their index pregnancy as unintended (not wanted at all or mistimed) compared with women reporting no previous induced abortion (64 %).

**Table 2:** Reproductive profile (%) of young women (12–24 years) seeking abortion-care services

	No previous induced abortion	Previous induced abortion	All young women
	*n* = 1,280 (91 %)	*n* = 98 (9 %)	*N* = 1,378
Parity			
0	63	62	63
1	22	26	23
2+	15	12	15
Type of contraception used (pre-abortion)*			
None	77	53	75
Pill	5	14	5
Injection/implant/IUD	9	5	9
Male condom	2	3	2
Emergency contraception	5	20	6
Rhythm/withdrawal/LAM	2	5	2
Pregnancy desired*			
Then	34	7	31
Later	31	33	31
Not at all	33	49	34
Unsure	3	10	3
Gestational age of index pregnancy			
12 weeks or less	62	51	61
13 weeks or more	36	45	37
Unknown	2	4	2
Severity of complications			
Low	30	29	30
Moderate	37	23	36
Severe	32	48	34
Contraception used (post-abortion)^†^			
Provided a modern method of contraception	53	72	55
Referred for contraceptive services	18	11	17
No contraception nor referral	29	17	28
Type of contraception received (post-abortion)*			
None	49	32	48
Pill	15	11	15
Injection/implant/IUD/sterilization/patch	30	44	32
Male or female condom	6	12	6

^†^ *p* < 0.10; *p < 0.05 for differences between young women based on previous history of induced abortion based on chi-square tests

Overall, 55 % of young women receiving abortion-related care received a modern method of contraception while 17 % received a referral for contraceptive services. The proportion of women receiving a modern method of contraception after treatment was greater among women reporting a previous induced abortion (72 %) compared with those reporting no prior abortion (53 %). However, the difference in proportions was only marginally statistically significant.

Results of the logistic regression models that assessed the factors associated with the likelihood of reporting a previous induced abortion are summarized in Table 3. At bivariate level (Model 0), all the socio-demographic and reproductive variables were significantly associated with the outcome variable. In the multivariable model comprising only socio-demographic variables (Model 1), age and marital status were not significantly associated with the likelihood of reporting a previous induced abortion. Rural residents were significantly less likely to report a previous induced abortion than urban residents. At bivariate level, young women with no education or incomplete primary education were more likely to report a previous induced abortion than those with higher education. Although similar associations between education and the outcome variable were observed in the model controlling for other socio-demographic characteristics, the difference was only statistically significant for young women having a post-secondary education compared with those with no or incomplete primary education. With respect to occupational status, at bivariate level, compared with those who reported that they were unemployed/other, respondents reporting all other occupations were less likely to report a previous induced abortion. After controlling for other socio-demographic characteristics (model 1), the difference was only significant for housewives compared with those who reported that they were unemployed/other. This association remained significant when reproductive variables were added (Model 2).

**Table 3:** Logistic regression models predicting the likelihood of reporting a previous induced abortion among young women

	Model 0			Model 1^a^			Model 2^a^		
	OR _unadjusted_	95 % Confidence Interval		OR _adjusted_	95 % Confidence Interval		OR _adjusted_	95 % Confidence Interval	
		Lower	Upper		Lower	Upper		Lower	Upper
*Socio-demographics*									
Age group									
10–19 years (ref)	--			--			--		
20–24 years	0.09*	0.06	0.15	0.82	0.44	1.55	0.90	0.42	1.95
Residence									
Urban (ref)	--			--			--		
Rural	0.05*	0.03	0.11	0.21*	0.07	0.61	0.28*	0.11	0.70
Education									
No education/incomplete primary (ref)	--			--			--		
Complete primary	0.13*	0.06	0.27	1.35	0.52	3.47	1.22	0.43	3.48
Incomplete secondary	0.12*	0.05	0.28	0.75	0.25	2.23	0.82	0.31	2.18
Complete secondary	0.13*	0.07	0.22	0.64	0.28	1.47	0.64	0.23	1.77
Post-secondary	0.08*	0.04	0.14	0.22*	0.07	0.68	0.21*	0.07	0.67
Marital status									
Never married (ref)	--			--			--		
Currently/formerly married	0.07*	0.03	0.15	0.83	0.40	1.68	0.72	0.31	1.65
Religion									
Roman Catholic (ref)	--			--			--		
Other Christian	0.09*	0.05	0.15	0.42*	0.22	0.78	0.42*	0.21	0.85
Muslim	0.01*	0.00	0.05	0.08*	0.02	0.29	0.09*	0.02	0.37
Other/No religion	0.40	0.11	1.49	4.63*	1.10	19.40	4.65*	1.03	20.95
Occupation									
Unemployed/other (ref)				--			--		
Housewife	0.01*	0.00	0.05	0.09*	0.03	0.27	0.08*	0.03	0.25
Student	0.13*	0.08	0.22	0.94	0.36	2.46	0.94	0.30	2.92
Skilled/clerical/sales/services	0.15*	0.07	0.33	0.85	0.35	2.06	0.75	0.30	1.86
Farmer/unskilled laborer	0.15*	0.06	0.41	0.86	0.33	2.23	0.93	0.38	2.29
*Reproductive characteristics*									
Parity									
0 (ref)	--						--		
1	0.11*	0.06	0.22				0.94	0.40	2.23
2+	0.07*	0.02	0.23				0.88	0.33	2.38
Type of contraception used (pre-abortion)									
None (ref)	--						--		
Pill	0.29*	0.09	0.92				3.35^†^	0.94	11.93
Injection/implant/IUD	0.05*	0.01	0.27				0.75	0.11	4.90
Male condom	0.14*	0.04	0.55				1.78	0.37	8.68
Emergency contraception	0.37^†^	0.13	1.04				3.03*	1.02	9.06
Rhythm/withdrawal/LAM	0.27*	0.12	0.63				3.30^†^	0.96	11.37
Pregnancy desired									
Then (ref)	--						--		
Later	0.10*	0.05	0.19				0.56	0.22	1.44
Not at all	0.14*	0.09	0.23				0.61	0.27	1.36
Unsure	0.35	0.09	1.34				1.31	0.46	3.76

^†^ *p* < 0.10 and *p < 0.05 (based on adjusted Wald tests); ref = reference group

^a^The adjusted odds ratios reported here were derived from the multivariable logistic regression analysis that contained all the socio-demographic (Model 1) and all the socio-demographic and reproductive characteristics (Model 2) shown in the table

The type of contraception used prior to the index pregnancy was the only reproductive variable significantly associated with the likelihood of reporting a previous induced abortion. Specifically, respondents who reported the use of emergency contraception were about three times more likely than those reporting no contraceptive use to report a previous induced abortion. Those reporting that they had used the oral pill and those reporting the use of the rhythm method, withdrawal or lactational amenorrhea were more likely to report a previous induced abortion compared with those reporting no contraceptive use. However, the differences between non-users and those reporting the use of the oral pill, or the rhythm method, withdrawal and lactational amenorrhea were only marginally significant (level of significance < 10 %)

## Discussion

Having multiple induced abortions, in contexts where safe abortion is restricted, can exacerbate health risks for women. However, there has been minimal interrogation of the prevalence of and factors associated with reporting a previous induced abortion among young women receiving abortion-related care in sub-Saharan Africa. In this study, we examined the factors associated with the likelihood of reporting a previous induced abortion among young women aged 12–24 years presenting for abortion or post-abortion related treatment in selected facilities in Kenya. About one out of every ten study participants reported a previous induced abortion. Area of residence (urban versus rural), education, religion, employment, and contraceptive use at the time of the index pregnancy were significantly associated with the likelihood of reporting a previous induced abortion. Marital status, parity and pregnancy intentions were not associated with the likelihood of reporting a previous induced abortion. Further, in contrast to previous studies on multiple abortions [14–16], we found no significant association between the likelihood of reporting a previous induced abortion and age, when controlling for other socio-demographic and reproductive characteristics of young women. While research by Tietze [13] in the United States suggests that the likelihood of having multiple induced abortions would increase with age and parity, we posit that in a context where the majority of induced abortions are unsafe, these associations may be more difficult assess because of: one, the high mortality associated with unsafe abortions; and two, the stigma associated with abortion, which may limit women’s willingness to report a previous induced abortion.

Our data showed that young urban dwellers had a greater likelihood of reporting a previous induced abortion compared with their rural counterparts. Previous research [25–27] demonstrates that young women in urban areas, particularly those in resource-limited areas, begin having sexual intercourse earlier and are more likely to have multiple sexual partners compared with their peers in rural and wealthier urban settings. These sexual behaviors are associated with a greater likelihood of having multiple abortions [17]. However, it is also important to note that the greater odds of reporting a previous induced abortion among urban women may also reflect greater access to health care in urban contexts and, possibly, higher probability of surviving a previous abortion.

The likelihood of reporting a previous induced abortion differed by level of education. Specifically, women with no education or incomplete primary education were over four times more likely to report a previous induced abortion compared with women with secondary or higher education. Similarly, in the United Kingdom, Stone and Ingham [17] found that women with lower levels of education were more likely to report multiple abortions. A high level of education has consistently been associated with greater use of contraception and lower unmet need for contraception [28]. As such, highly educated women may be less likely to have unintended pregnancies and consequently, induced abortions. Further, the link between higher education and greater incomes [29] suggests that more educated women may have the financial ability to take care of children whose birth was unintended or to have access to safe abortion services that may offer better post-abortion contraceptive counseling. The long term and wider health benefits of higher education suggest that interventions that address more distal determinants of health, such as access to education, can have a direct and sustainable impact on improving youth sexual and reproductive health.

We found that a greater proportion of young women reporting a previous induced abortion stated that they were using a contraceptive method (albeit methods with high failure rates) at the time of the occurence of the index pregnancy compared with those reporting no previous induced abortion. A greater proportion of respondents reporting a previous induced abortion also described their index pregnancy as unintended compared with those reporting no previous induced abortion. Higher levels of contraceptive use among young women having a second or third abortion compared with women having their first abortion have also been reported in Norway [30] and the United States [31]. As Cohen [31] notes, the greater use of contraceptives among women reporting a previous abortion casts doubt on assumptions that many women rely on abortion as a means to prevent unplanned births. Instead, these results suggest that women who have had a previous abortion may instead be more likely to use contraception to avoid unintended pregnancy but may need counseling and access to a wider range of effective contraceptive methods to minimize chances of contraceptive failure.

The finding that women reporting a previous induced abortion were more likely to receive post abortion contraception, particularly long-acting methods, is encouraging. However, our results suggest that a substantial proportion of women may continue to be at risk of subsequent unintended pregnancies and consequently, unsafe abortion because they do not receive an effective contraceptive method after an abortion. As such, efforts to improve the quality of post-abortion contraception counseling and services are critical. Previous research suggests that immediate provision of long-acting methods, in particular, during post-abortion treatment may be highly effective in reducing the likelihood of subsequent abortions [20].

### Limitations

Our findings must be interpreted in light of several limitations. First, the stigma surrounding abortion in Kenya, as in other parts of sub-Saharan Africa, means that reporting of abortion may be subject to social desirability bias. Studies conducted in countries with more liberal laws around abortion have documented higher levels of multiple induced abortions among young women [15–19, 21]. Second, the data reported here are based on information collected from young women who presented to a facility primarily for post-abortion care. Given the high risk of mortality associated with unsafe abortion and the stigma associated with abortion care, it is plausible that the numbers presented here underestimate the true prevalence of multiple induced abortions. Third, causal inferences cannot be made due to the cross-sectional nature of the study. Finally, while the study allows us to examine an array of individual-level factors that may increase the likelihood of multiple induced abortions among young women in Kenya, the original study was not designed to measure a wide range of factors at individual, family or community level that would enable a more nuanced assessment of possible correlates of having multiple induced abortions. Nonetheless, the current study makes a significant contribution to existing knowledge around multiple induced abortions among young women. A key strength of this paper is the use of data from a relatively large sample of young women seeking abortion-related care at a nationally-representative sample of health facilities in the country.

## Conclusion

Our study results show that about one in every ten young women seeking abortion-related care in Kenya has had a previous induced abortion. While studies conducted in countries with more liberal laws around abortion have reported higher levels of multiple induced abortions among young women [15–19, 21], the proportion of young women reporting a previous induced abortion in our study, while low, is not trivial because it is likely to be an under-estimate given restrictions around safe abortion service provision and the stigma associated with abortion in the Kenyan context. Limited access to youth-friendly comprehensive post-abortion care may mean that young women who have had an abortion – whether safe or unsafe – may be at significant risk for future unintended pregnancies and, consequently, subsequent abortions. Investments in ensuring access to comprehensive post-abortion care services targeting young women are therefore needed. In particular, post-abortion care health service providers must ensure that young clients receive adequate contraceptive counseling and access to a range of effective contraceptive methods. Concurrently, investments in distal determinants of health, such as access to schooling and improved livelihoods, may have a sustainable impact on improving youth sexual and reproductive health.

## Ethics

The study protocol was reviewed and approved by the Ethical Review Boards of the Kenya Medical Research Institute, the University of Nairobi/ Kenyatta National Hospital, Moi University Teaching and Referral Hospital, Kenya, and Aga Khan University, Kenya. The Ministries of Public Health and Sanitation and Medical Services in Kenya and the Institutional Review Board of the Guttmacher Institute also reviewed and approved the study protocol.

## Consent to participate

Verbal informed consent was obtained from all women presenting for care (or their caregivers) prior to data collection.

## Consent to publish

Not applicable.

## Availability of data and materials

The data used in this study can be accessed via the APHRC Microdata Portal (http://aphrc.org/catalog/microdata/index.php/catalog/39).
